# Antidepressant Use and Suicide Rates in Adults Aged 75 and Above: A Swedish Nationwide Cohort Study

**DOI:** 10.3389/fpubh.2021.611559

**Published:** 2021-02-19

**Authors:** Khedidja Hedna, Johan Fastbom, Annette Erlangsen, Margda Waern

**Affiliations:** ^1^Department of Psychiatry and Neurochemistry, Centre for Aging and Health (AGECAP), Gothenburg University, Gothenburg, Sweden; ^2^Statistikkonsulterna AB, Gothenburg, Sweden; ^3^Aging Research Center, Karolinska Institute and Stockholm University, Stockholm, Sweden; ^4^Mental Health Centre, Danish Research Institute for Suicide Prevention, Copenhagen, Denmark; ^5^Department of Mental Health, Johns Hopkins Bloomberg School of Public Health, Baltimore, MD, United States; ^6^Centre for Mental Health Research, Research School of Population Health, The Australian National University, Canberra, ACT, Australia; ^7^Psychosis Clinic, Sahlgrenska University Hospital, Region Västra Götaland, Gothenburg, Sweden

**Keywords:** suicide, antidepressants, pharmacoepidemiology, drug utilization, older adults

## Abstract

**Background:** The treatment of depression is a main strategy for suicide prevention in older adults. We aimed to calculate suicide rates by antidepressant prescription patterns in persons aged ≥ 75 years. A further aim was to estimate the contribution of antidepressants to the change in suicide rates over time.

**Methods:** Swedish residents aged ≥ 75 years (*N* = 1,401,349) were followed between 2007 and 2014 in a national register-based retrospective cohort study. Biannual suicide rates were calculated for those with selective serotonin reuptake inhibitor (SSRI) single use, mirtazapine single use, single use of other antidepressants and use of ≥ 2 antidepressants. The contribution of antidepressants to the change in biannual suicide rates was analyzed by decomposition analysis.

**Results:** There were 1,277 suicides. About one third of these were on an antidepressant during their last 3 months of life. In the total cohort, the average biannual suicide rate in non-users of antidepressants was 13 per 100,000 person-years. The corresponding figure in users of antidepressants was 34 per 100,000 person-years. These rates were 25, 42 and 65 per 100,000 person-years in users of SSRI, mirtazapine and ≥ 2 antidepressants, respectively. In the total cohort, antidepressant users contributed by 26% to the estimated increase of 7 per 100,000 in biannual suicide rates. In men, biannual suicide rates increased by 11 suicides per 100,000 over the study period; antidepressant users contributed by 25% of the change. In women, those on antidepressant therapy accounted for 29% of the estimated increase of 4.4 per 100,000.

**Conclusion:** Only one third of the oldest Swedish population who died by suicide filled an antidepressant prescription in their last 3 months of life. Higher suicide rates were observed in mirtazapine users compared to those on SSRIs. Users of antidepressants accounted for only one quarter of the increase in the suicide rate. The identification and treatment of suicidal older adults remains an area for prevention efforts.

## Introduction

The oldest segment of the population is accountable for the highest suicide rates in many countries (1). Depression constitutes a major risk factor in persons aged 75 and above (2). Thus, identification and treatment of depression is a central strategy for suicide prevention in this age group.

Currently, over one fifth of the Swedish population aged 75 and above is prescribed antidepressants (ADs), mainly SSRIs and mirtazapine (3). Antidepressants have varied effects on pathophysiology; they differ in their impact on anxiety and sleep as well as the likelihood of adverse discontinuation effects (4). Meta-analyses of controlled trials have indicated possible differences in risk of suicidal behavior among different ADs (5, 6). Some research has found a higher suicide risk for persons initiating treatment with SSRI compared to other AD (7). Other studies reported lower rates of suicidal behavior in those treated with SSRI compared to other ADs (8), while those treated with mirtazapine had the highest suicide rates (8–10). Yet, others reported no difference (11). Such mixed results may be expected, given that suicide is a rare event and studies examined divergent populations and prescribing patterns. It remains unclear to what extent suicide rates vary in older people treated with different ADs (12, 13), or in those using multiple AD medications, which is a main therapeutic option for those with inadequate response to monotherapy (14).

Some previous research suggested an association between the increase in AD use and a decrease in suicide rates (13, 15, 16). However, the body of evidence was either mainly based on mixed age groups in ecological studies without demonstrating a direct relationship at the individual level (13, 15, 16), included younger age groups (9), or younger older adults (17). Therefore, the contribution of AD to the change in the suicide rates among those aged 75 and above warrants further examination. A Danish study used decomposition techniques to calculate the contribution of AD use to the change in the suicide rate among individuals aged 50+ (10). It found that individuals in active treatment with antidepressants accounted for only 10% of the change in the suicide rate. Findings cannot be extrapolated to the oldest segment of the population as patterns of AD use and treatment response may differ, due to higher levels of age-related psychiatric and somatic comorbidities, as well as physiological changes (18).

The use of nationwide, register-based data can accrue the large sample sizes needed to examine suicide deaths with sufficient power and minimize the risk of selection bias. We aimed to investigate suicide rates in adults aged 75 years and above for the most common antidepressant prescription patterns. A further aim was to estimate the contribution of antidepressants to the change in suicide rates over time.

## Method

### Study Design and Study Population

We conducted a retrospective cohort register-based study of all Swedish residents aged 75 years and older between January 1, 2007 and December 31, 2013. Individuals had to be recorded as living in Sweden during the year prior to study entry in order to be included and were followed until December 31, 2014 or censored in case of death due to causes other than suicide.

### Data Sources

Data from various registers were linked using the personal identity number. All Swedish residents aged 75 years and older during the study period were identified from the Total Population Register at Statistics Sweden. The Swedish Prescribed Drug Register was employed to capture AD use. The register has full coverage of dispensed prescriptions in outpatient care and in residential care (19). It includes information on dispensed medications, dispensed quantity, number of daily doses and the type of prescription. The Swedish Cause of Death Register includes data on age, sex, date of death and the International Classification of Diseases (ICD) codes for underlying and contributing causes of death including suicide (20). These registers are held by the National Board of Health and Welfare.

### Exposure to Antidepressants

First, we identified individuals who were exposed to AD (Anatomical Therapeutic Chemical group: N06A) during the year that preceded the suicide and recorded the date of last refill and the specific AD prescribed (Supplementary Material 1). We excluded from the analyses persons taking tricyclic antidepressants (TCA) without any other AD (*n* = 46,244), in order to reduce confounding by indication. The rationale for this was that these medications are no longer recommended for the treatment of depression in older adults in Sweden and are instead used for treatment of chronic pain (21).

In order to ensure sufficient power, we considered categories of the prescription patterns most commonly seen in older adults in Sweden: (a) single use of SSRI, (b) single use of mirtazapine, (c) single use of other AD, and (d) use of ≥ 2 different ADs.

The use of AD was considered as a time-varying exposure. In Sweden, patients may purchase up to 3 months' supply when redeeming a prescription. Alternatively, they may use the multidose system, which provides an automatic refill every 2 weeks. A person was considered as being on continuous exposure to AD treatment if she/he had ≥ 3 refills of AD – or ≥ 20 automatic refills for those using multidose – over a 12-month period. This approach ensured that AD medication covered 75% of days as a minimum. For individuals who died during the observation period, we calculated the proportion of days covered by AD during their last year of life to determine whether they should be considered to be on AD therapy.

### Characteristics of the Study Population

We collected data as recorded during the year participants entered the cohort. Characteristics included: sex, age group (75–79, 80–84, 85–89, and ≥90 years), previous episode of self-harm, residence in institution, use of other psychoactive medications (anxiolytics, hypnotics, antipsychotics, anti-dementia, mood stabilizers) and use of cardiovascular medications (beta-blockers and statins). The selected characteristics were previously found to be associated with both antidepressant use and suicide. The use of specialized care for depression was considered as a proxy for serious depression. The relationship of the selected variables with suicide in users and non-users of antidepressant older than 75 years was previously investigated in our published study (22).

### Study Outcomes

Suicides were identified from the Cause of Death Register based on ICD-10 codes: Intentional self-harm (X60-X84), harm of undetermined intent (Y10-Y34), and sequelae of intentional self-harm and of events of undetermined intent (Y87.0 and Y87.2).

### Statistical Analysis

First, we compared the baseline characteristics of users and non-users of AD using *t*-test for continuous variables and Pearson's chi square test for categorical variables. The latter test was also employed to test for differences in proportions of individuals using other psychoactive medications among AD user groups compared with those using solely SSRI.

In order to distinguish random fluctuations due to small numbers from true changes, biannual suicide rates were calculated by AD use group and period for all AD users and non-users as well as for the most commonly prescription patterns.

A decomposition technique was used to calculate the contribution of users of antidepressants to the change in the overall suicide rate. The decomposition technique is a method derived from demographic studies, widely used in health and behavior studies when rates of a phenomenon within the same population are compared at two different time points. This method takes into account the difference in the population composition between the time points in order to adjust for the confounding effect of population composition on the measured rates and accounts for the impact of compositional factors on the observed rates (23). In our study, the decomposition method distinguishes between antidepressant treatment group-specific change and population-specific change (e.g., structural change related to different population counts by age and year) between two given time points. It is based on the proportion of person-years and the suicide rates by antidepressant treatment category measured for the two time-periods. The overall suicide rate is equal to the sum of each treatment group's suicide rate multiplied by the proportion of person-years spent in that group at that time. The two components are used in two ways: mean weights and the difference of proportions. The first covers changes in the overall suicide rate attributed to changes in the antidepressant treatment group-specific suicide rate and the second component accounts for changes in the population composition. The total change in suicide rate equals the sum of the antidepressant treatment group-specific and population-specific changes. Results were calculated first for the total population and then by gender. Equations employed in the decomposition technique are provided in Supplementary Material 2, along with an example calculation. Data analyses were performed by SAS version 9.4 (SAS Institute Inc, NC, USA).

As the analyses were based on existing population register data, informed consent from subjects was not required. Approval was granted by register holders. Statistics Sweden replaced the Personal identity number by a study number prior to data delivery and data were analyzed anonymously. The study was approved by the Regional Ethical Review Board in Gothenburg (Ethical approval No: 111-15).

## Results

A total of 1,401,349 individuals (593,676 men and 807,673 women) were included in the cohort and followed for up to 8 years. This corresponds to 7,191,803 person-years (men, 2,931,665; women, 4,260,139). Key baseline characteristics are presented by AD use in Table 1. AD users were slightly older than non-users. Higher proportions of women, as well as residents in institutions were observed among AD users. The most commonly used ADs were SSRI followed by mirtazapine; users of mirtazapine were more likely to have other psychoactive medications than users of SSRI. Use of specialized care for depression, a marker of a more serious level of depression, was the highest in users of ≥ 2 ADs.

**Table 1:** Baseline characteristics of persons aged 75 and above residing in Sweden between 2007 and 2014^a^ (*N* = 1,401,349).

	**Non-user of antidepressants at baseline**	**Users of antidepressants at baseline**				
	**(*N* = 1,263,761)** ***n* (%)^b^**	**Total (*N* = 137,588)** ***n* (%)**	**Single use of SSRI (*N* = 95,929) *n* (%)**	**Single use of mirtazapine (*N* = 19,811)** ***n* (%)**	**Single use of other antidepressants (*N* = 10,451)** ***n* (%)**	**Use of ≥ 2 antidepressants (*N* = 11,173)** ***n* (%)**
**Age**						
Mean (SD)^c^	79.9 (5.8)^***^	81.2 (6.0)	81.4 (6.1)	81.5 (6.1)	79.5 (5.5)	80.7 (5.8)
Median (min-max)	78 (75–112)	80 (75–106)	80 (75–106)	81 (75–106)	77 (75–103)	79 (75–103)
75-79	734,598 (58.1)	65,391 (47.5)	44,285 (46.2)	8,995 (45.4)	6,356 (60.8)	5,644 (50.5)
80-84	249,726 (19.8)	30,666 (22.3)	21,642 (22.6)	4,426 (22.3)	2,025 (19.4)	2,522 (22.6)
85-89	174,768 (13.8)	26,500 (19.3)	18,988 (19.8)	4,053 (20.5)	1,438 (13.8)	1,983 (17.7)
90+	104,669 (8.3)	15,031 (10.9)	11,014 (11.5)	2,337 (11.8)	632 (6)	1,024 (9.2)
Women^d^	710,499 (56.2)^***^	97,174 (70.6)	68,104 (71)	13,633 (68.8)	7,463 (71.4)	7,815 (69.9)
**Use of other psychoactive medications^e^**						
Hypnotics	331,863 (26.3)	76,750 (55.8)	50,177 (52.3)	12,796 (64.6)^***^	6,301 (60.3)^***^	7,335 (65.6)^***^
Anxiolytics	189,919 (15)	65,144 (47.3)	40,692 (42.4)	11,450 (57.8)^***^	5,441 (52.1)^***^	7,441 (66.6)^***^
Antipsychotics	52,891 (4.2)	22,925 (16.7)	13,048 (13.6)	4,582 (23.1)^***^	2,079 (19.9)^***^	3,192 (28.6)^***^
Anti-dementia	31,127 (2.5)	14,679 (10.7)	10,197 (10.6)	2,383 (12)^***^	710 (6.8)^***^	1,366 (12.2)^***^
Mood stabilizers	19,518 (1.5)	6,266 (4.6)	3,722 (3.9)	899 (4.5)^***^	758 (7.3)^***^	895 (8.0)^***^
**Use of cardiovascular medications**						
Beta-blockers	501,731 (39.7)	56,659 (41.2)	40,135 (41.8)	8,214 (41.5)	4,052 (38.8)^***^	4,152 (37.2)^***^
Statins	343,261 (27.2)	37,493 (27.3)	26,811 (27.9)	4,748 (24)^***^	3,073 (29.4)^**^	2,792 (25)^***^
Diagnosis of depression in hospital or specialized outpatient care	6,853 (0.5)^***^	12693 (9.2)	5,282 (5.5)	2,718 (13.7)	1,629 (15.6)	3,046 (27.3)

a *Excluding users of tricyclic antidepressants with no other AD prescription (N = 46,244)*.

b *Compared with- all users of antidepressant at baseline*.

c *Two sample t-test assuming unequal variance*.

d *Pearsons Chi^2^-test*.

e *Chi^2^-test for comparison with single use of SSRI*.

** *P ≤ 0.01*.

*** *P ≤ 0.001*.

In the total group, there were 1,277 suicides during the study period, and 70% of these occurred among men. Overall, 404 (31.6%) filled at least one AD during their final 3 months of life (28.6% of men and 38.6% of women) (Table 2).

**Table 2:** Distribution of suicide by exposure to and type of (non-tricyclic) antidepressant as well as time since the last antidepressant fill in persons aged 75 and above residing in Sweden between 2007 and 2014.

	**Total** ** (*N* = 1,277)** ** (%)**	**Men** ** (*N* = 894)^a^** ** (%)**	**Women** ** (*N* = 383)^b^** **(%)**
**Time since last prescription before suicide**			
≤ 1 year	492 (38.5)	311 (34.8)	181 (47.3)
≤ 6 months	453 (35.5)	285 (31.9)	168 (43.9)
≤ 3 months	404 (31.6)	256 (28.6)	148 (38.6)
≤ 1 month	306 (24.0)	197 (22.0)	109 (28.5)
≤ 1 week	128 (10.0)	84 (9.4)	44 (11.5)
**Type of antidepressants**			
Single use of SSRI	209 (16.4)	129 (14.4)	80 (20.9)
Single use of citalopram	129 (10.1)	85 (9.5)	44 (11.5)
Single use of sertraline	48 (3.8)	28 (3.1)	20 (5.2)
Single use of escitalopram	18 (1.4)	10 (1.1)	8 (2.1)
Single use of mirtazapine	95 (7.4)	64 (7.2)	31 (8.1)
Single use of other antidepressants	18 (1.4)	12 (1.3)	6 (1.6)
Use of ≥ 2 antidepressants	169 (13.2)	120 (13.4)	63 (16.4)

*SSRI, Selective Serotonin Reuptake Inhibitor*.

a *Represent 70% of total suicides*.

b *Represent 30% of total suicides*.

**Figure 1:**
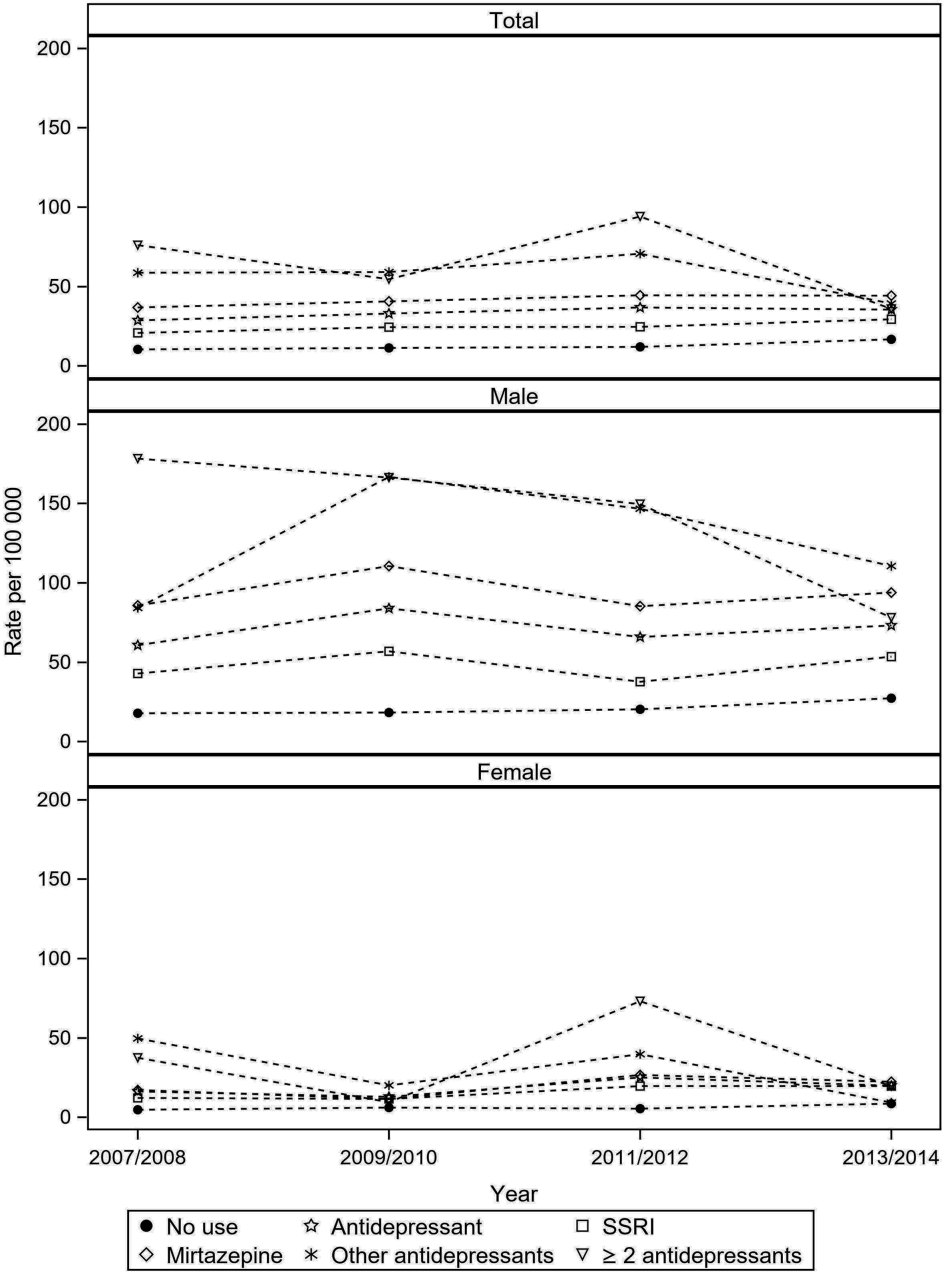
Biannual suicide rate by in antidepressant users and non-users among residents aged 75 and above in Sweden from 2007 to 2014^a^. ^a^Excluding users of tricyclic antidepressants with no other antidepressant prescription (*N* = 46,244).

In the total cohort, the average biannual suicide rates in those who were not on AD was 13 per 100,000 person-years (Figure 1). The corresponding figure in those who were on AD was 34 per 100,000 person-years. The average rate in those who used SSRI was 25 per 100,000 person-years; the corresponding figure was 42 per 100,000 for those who used mirtazapine as single AD. For those with ≥ 2 AD, the suicide rate was 65 per 100,000. The corresponding figure in those who were not on AD was 13 per 100,000 person-years. The average biannual suicide rate for older men who were on AD was 71 per 100,000 person-years. The average rate in men who used SSRI was 48 per 100,000 person-years; the corresponding figure was 94 per 100,000 for those who used mirtazapine as single AD. For men with ≥ 2 AD, the suicide rate was 143 per 100,000. The suicide rate for men not treated with AD was 21 per 100,000 person-years. For women, the average suicide rate for those who used AD was 19 per 100,000 person-years. The average rate in women how used SSRI was 16 per 100,000 person-years. The corresponding figure in users of mirtazapine was 19 per 100,000. For women with ≥ 2 AD, the suicide rate was 35 per 100,000. Women not on AD treatment had a suicide rate of 6 per 100,000 person-years. Suicide rates per each AD category were found to vary over time due to few observations (Supplementary Material 3).

During the study period, in the total cohort, the percentage of person-years spent in AD treatment increased from 8.1% in 2007–2008 to 11.7% in 2013–2014 and the overall suicide rate increased from 12 to 19 suicides per 100,000 during the same period (Table 3). In men, the percentage of person-years spent in AD treatment increased from 5.3 to 8.1% and the suicide rate increased by 11 per 100,000 from 20.1 to 31.1. In women, the percentage of person-years spent in AD treatment increased from 10.2 to 14.3%, and the suicide rate increased by 4.3 per 100,000 from 5.9 to 10.2 per 100,000 over the same period.

**Table 3:** Distribution of biannual suicide rate per 100,000 person-years by antidepressant treatment group among persons aged 75 and above residing in Sweden^a^ for 2007–2008 and 2013–2014.

**Characteristics**	**Person-years (%)**		**N suicide (%)**		**Suicide rate per 100,000 (95% CI)**	
	**2007–2008**	**2013–2014**	**2007–2008**	**2013–2014**	**2007–2008**	**2013–2014**
**Total**						
No antidepressant use	2,326,127 (91.9)	1,652,154 (88.3)	244 (80.5)	278 (78.1)	10.5 (9.3–11.7)	16.8 (15.1–18.6)
Antidepressant use^b^	205,428 (8.1)	219,626 (11.7)	59 (19.5)	78 (21.9)	28.7 (25.6–31.9)	35.5 (31.8–39.2)
Single use of SSRI	149,141 (5.9)	136,317 (7.3)	31 (10.2)	40 (11.2)	20.8 (18.4–23.1)	29.3 (26.2–32.5)
Single use of mirtazapine	32,549 (1.3)	51,895 (2.8)	12 (4)	23 (6.5)	36.9 (32.8–41)	44.3 (39.8–48.8)
Single use of other antidepressants	13,630 (0.5)	15,259 (0.8)	8 (2.6)	6 (1.7)	76 (67.4–84.6)	35.8 (32–39.5)
Use of ≥ 2 antidepressants	18,426 (0.7)	27,955 (1.5)	14 (4.6)	10 (2.8)	58.7 (52–65.4)	39.3 (35.2–43.4)
Total	2,531,555 (100)	1,871,780 (100)	303 (100)	356 (100)	12 (10.6–13.3)	19 (17.1–21)
**Men**						
No antidepressant use	1,025,564 (94.7)	723,378 (91.9)	183 (83.9)	198 (80.8)	17.8 (15.5–20.2)	27.4 (23.8–30.9)
Antidepressant use^b^	57,589 (5.3)	64,185 (8.1)	35 (16.1)	47 (19.2)	60.8 (52.7–68.8)	73.2 (64–82.4)
Single use of SSRI	41,895 (3.9)	39,176 (5)	18 (8.3)	21 (8.6)	43 (37.3–48.6)	53.6 (46.9–60.3)
Single use of mirtazapine	9,336 (0.9)	15,984 (2)	8 (3.7)	15 (6.1)	85.7 (74.3–97.1)	93.8 (82.1–105.6)
Single use of other antidepressants	3,560 (0.3)	4,524 (0.6)	3 (1.4)	5 (2)	178.3 (154.5–202)	78.1 (68.3–87.9)
Use of ≥ 2 antidepressants	5,049 (0.5)	7,686 (1)	9 (4.1)	6 (2.4)	84.3 (73.1–95.4)	110.5 (96.6–124.4)
Total	1,083,153 (100)	787,564 (100)	218 (100)	245 (100)	20.1 (17.4–22.9)	31.1 (27.2–35)
**Women**						
No antidepressant use	1,300,564 (89.8)	928,776 (85.7)	61 (71.8)	80 (72.1)	4.7 (3.7–5.7)	8.6 (7–10.2)
Antidepressant use^b^	147,838 (10.2)	155,440 (14.3)	24 (28.2)	31 (27.9)	16.2 (12.7–19.8)	19.9 (16.2–23.7)
Single use of SSRI	107,246 (7.4)	97,141 (9)	13 (15.3)	19 (17.1)	12.1 (9.6–14.7)	19.6 (15.8–23.3)
Single use of mirtazapine	23,213 (1.6)	35,911 (3.3)	4 (4.7)	8 (7.2)	17.2 (13.5–21)	22.3 (18.2–26.4)
Single use of other antidepressants	10,070 (0.7)	10,735 (1)	5 (5.9)	1 (0.9)	37.4 (29.3–45.4)	19.7 (16–23.5)
Use of ≥ 2 antidepressants	13,377 (0.9)	20,269 (1.9)	5 (5.9)	4 (3.6)	49.7 (39.1–60.2)	9.3 (7.6–11.1)
Total	1,448,402 (100)	1,084,217 (100)	85 (100)	111 (100)	5.9 (4.7–7)	10.2 (8.3–12.2)

a *Excluding persons using tricyclic antidepressants (ADs) and no other AD (N = 46,244)*.

b *Defined by ≥3 fills of ADs or ≥20 automatic fills for those using multidose during the specified year, corresponding to AD exposure 75% of the time. For new users, this definition was adjusted to the number of days since their index fill in the considered year. For individuals who died during the observation period, the number of AD refilled were calculated in their last year of life to determine whether they should be considered to be on AD therapy 75% of the time*.

*SSRI, Selective Serotonin Reuptake Inhibitor*.

As seen in Table 4, the decomposition analysis separated the total contribution of each treatment group to the change in the suicide rate (column 3) into two components: the change in the treatment-specific suicide rate (column 1) and the change in the population composition (column 2). In the total cohort, AD non-users accounted for 5.2 out of 7 suicides per 100,000, equivalent to 74% of the increase, while those who were AD users accounted for the remaining 1.8 suicides per 100,000, corresponding to 26% of the increase. The slight increase in the number of AD users during the study period had a small contribution to the overall change in the suicide rate in the population aged 75 and above. Men who were not on AD accounted for 8.3 suicides per 100,000 of the total increase in the male suicide rate of 11 suicides per 100,000; equivalent to 75% of the increase in the suicide rate over the observation period. Men who were AD users contributed with 2.7 suicide per 100,000 to the observed total increase or a share of 25%. Women who were not on AD treatment accounted with 3.2 out of 4.4 suicides per 100,000, equivalent to 72% of the increase, while those who were AD users accounted for the remaining 1.2 suicides per 100,000, corresponding to 28% of the increase.

**Table 4:** Decomposition of the change over time in the suicide rate per 100,000 by antidepressant (AD) treatment group among persons aged 75 and above residing in Sweden^a^ from 2007–2008 to 2013–2014.

**Population**	**Change in suicide rate attributed to changes in treatment group-specific suicide rate**	**Change in suicide rate attributed to changes in population composition**	**Total contribution to change in suicide rate**
**Total**			
No antidepressant use	5.67	−0.49	5.18
Antidepressant use^b^	0.67	1.16	1.83
Single use of SSRI	0.56	0.35	0.91
Single use of mirtazapine	0.15	0.60	0.75
Single use of other antidepressants	−0.13	0.14	0.01
Use of ≥ 2 antidepressants	−0.45	0.43	−0.02
Total	6.34	0.67	7.01
**Men**			
No antidepressant use	8.95	−0.64	8.31
Antidepressant use^b^	0.83	1.9	2.73
Single use of SSRI	0.47	0.53	1
Single use of mirtazapine	0.12	1.05	1.17
Single use of other antidepressants	0.12	0.24	0.36
Use of ≥ 2 antidepressants	−0.72	0.65	−0.07
Total	9.78	1.26	11.04
**Women**			
No antidepressant use	3.42	−0.27	3.15
Antidepressant use^b^	0.45	0.75	1.2
Single use of SSRI	0.61	0.25	0.86
Single use of mirtazapine	0.13	0.34	0.47
Single use of other antidepressants	−0.34	0.09	−0.25
Use of ≥ 2 antidepressants	−0.25	0.27	0.02
Total	3.87	0.48	4.35

a *Excluding persons using tricyclic AD and no other AD (N = 46,244)*.

b *Defined by ≥3 refills of AD or ≥20 automatic refills for those using multidose during the specified year to ensure a proportion of days covered by AD therapy of 75%. For those who were followed less than a year, this definition was adjusted to the number of days the person was followed during the year to determine whether they should be considered to be on AD therapy*.

## Discussion

This nationwide cohort study covering Swedish residents aged 75 and above found that about one third of those who died by suicide filled an AD prescription during their final 3 months of life. Individuals who were on single AD treatment with mirtazapine, as well as those using more than one AD had higher suicide rates than those on a single SSRI. Older individuals not on AD therapy were accountable for the major share of the increase in the suicide rate during the study period while those on AD therapy made a small contribution to the overall change.

Only about one third of those who died by suicide in our national cohort of individuals aged 75 and above were on AD. Considering that two-thirds of persons over 75 who die by suicide may be depressed (2), there is reason to believe that a large proportion of suicides in this national cohort had untreated or undertreated depression at the time of death. It is possible that some clinicians identified depression but declined to prescribe ADs to their older patients due to some skepticism related to questions of efficacy, or possible concerns of emerging suicidality. Other potential issues involve side effects and the risk of withdrawal symptoms and/or increased symptom severity after cessation (24). Some older adults may be reluctant to seek mental healthcare due to their attitudes about mental conditions and negative beliefs about depression treatment (25). We note, however, that older adults with previous suicidal behavior experience that the benefits of AD treatment outweigh the disadvantages (26). Interventions that aim at optimizing depression treatment in primary care are promising (27), but our results suggest that programs that specifically target patients aged 75 and above need to be implemented.

Seemingly, no previous study has calculated suicide rates by type of AD in this age group. A Canadian population-based study conducted in a general adult population found suicide rates in the range of those in our population (11). In our study, suicide rates were the highest among individuals who used ≥ 2 antidepressants. This was also the group with the most extensive use of specialized healthcare for depression and who are likely to suffer from more severe depressions (28), and therefore, the finding is potentially being subject to confounding by indication. The same may explain the higher rates in users of mirtazapine as this AD may be prescribed to individuals who have not responded to the first choice AD treatment SSRI, which might reflect a more serious level of depression (29). Mirtazapine is also prescribed in the geriatric population for anxiety and sleep problems, both associated with increased risk of suicidal feelings in older populations (30, 31). The low suicide rates observed in those who were not on AD may in part reflect a lower prevalence of clinically relevant depression. We cannot test this as we lack individual level data on depressive symptomatology. In a smaller study using a case-control design, we found no increase in suicide risk among older adult users of antidepressants compared to non-users, once we adjusted for relevant psychiatric disorders (32).

Our decomposition analysis suggests that non-users of AD contributed the most to the change increase in the suicide rate, which may in part be explained by the role of other factors affecting suicide such as social factors and other comorbidities (33, 34). The exclusion of those who were exclusively on TCA and no other AD may have partially affected our findings but would hardly have a significant effect on the overall suicide rates as AD users had a small contribution to the change of suicide rates and those who were only under TCA represented only 3% of the whole 75 population.

### Methodological Considerations

This is to our knowledge the first study to calculate nationwide suicide rates for individuals aged 75 and above treated with AD. Complete and systematically assembled register data with no loss to follow-up are some of the strengths of this study. This large national cohort provided enough power to assess rates of a rare event like suicide, allowing us to decompose the contribution of ADs to the change in the suicide rate in the Swedish population aged 75 and above. A main limitation is the lack of diagnosis-specific data from primary care as this information is not available in the National Patient Register. We could therefore not adjust for confounding by indication. This is problematic as antidepressants may have been prescribed for conditions other than depression (anxiety, sleep disorders), also associated with suicidality in older adults even in the absence of depression (30, 31). Moreover, suicide rates may also be even higher in individuals with untreated mood disorders and with alcohol use disorders. However, our data set did not allow for the identification of untreated mental conditions and behavioral risk factors so we could not calculate their contribution to the change of the late-life suicide rate. Regarding the group with more than one AD prescription, the small numbers rendered it unfeasible to show separate suicide rates for those with longer-term concurrent prescriptions and those who had parallel prescriptions in connection with switching. However, both of these use patterns are markers of more severe depression.

We considered AD use as a time-varying exposure which allowed us to take into account changes in prescribing patterns that may have occurred during the 8-year study period. Our strict definition of continuous use of AD increased the likelihood that subjects had enough exposure to AD to derive therapeutic benefit. While we grouped biannual suicide rates to increase study power, the number of suicides for each specific substance (with the exception of mirtazapine) was small and did not allow for reasonable and stable estimation of suicide rates. The biannual rates in men and women should also be interpreted with caution due to small numbers. Comparison of our suicide rates with other countries should be interpreted with caution due to differences in population composition. Further, prescribing patterns may vary; some countries may still prescribe TCA for late-life depression. The results of this research may not be generalized to other countries or settings as availability of healthcare and reporting of suicide varies widely in a global perspective.

## Conclusion

Only one third of those aged 75 and above who died by suicide in this Swedish national cohort were on an antidepressant (non-tricyclic) during their last 3 months of life. Users of antidepressants accounted for only one quarter of the overall increase in the suicide rate in this age group between 2007 and 2014. Our findings, taken together with previous results demonstrating high rates of depression among older adults who die by suicide, highlight the need for intervention research to prevent suicide in this growing age group.

## Data Availability Statement

The data analyzed in this study is subject to the following licenses/restrictions: The datasets generated and/or analyzed during the current study are not publicly available due confidentiality but aggregated data are available from Statistics Sweden or the National Board of Health and Welfare upon request. Requests to access these datasets should be directed to https://scb.se/vara-tjanster/bestalla-mikrodata/, and https://www.socialstyrelsen.se/statistik-och-data/bestalla-data-och-statistik/.

## Ethics Statement

The studies involving human participants were reviewed and approved by Regional Ethical Review Board in Gothenburg (Ethical approval No: 111-15). Written informed consent for participation was not required for this study in accordance with the national legislation and the institutional requirements.

## Author Contributions

KH, MW, and JF designed the study. KH was the guarantor, coordinated the statistical analysis, and drafted the manuscript. KH, MW, JF, and AE interpreted the data and participated to the critical revision of the manuscript. All authors had full access to all of the data (including statistical reports and tables) in the study and can take responsibility for the integrity of the data and the accuracy of the data analysis.

## Conflict of Interest

The authors declare that the research was conducted in the absence of any commercial or financial relationships that could be construed as a potential conflict of interest.

## Supplementary Material

Supplementary Material 1 Figure visualizing antidepressant exposure patterns applied in a retrospective cohort study of adults aged 75 years and over.

Supplementary Material 2 Calculations of the decomposition method.

Supplementary Material 3 Biannual suicide rates per 100,000 (95% CI) by antidepressant treatment group for individuals aged 75 years and over in Sweden from 2007 and 2014.
