# Supplementary figures and images for: Antidepressant Use and Suicide Rates in Adults Aged 75 and Above: A Swedish Nationwide Cohort Study
Source: Front Public Health. 2021 Feb 19;9:611559. doi: 10.3389/fpubh.2021.611559 (PMC7933212; doi:10.3389/fpubh.2021.611559)

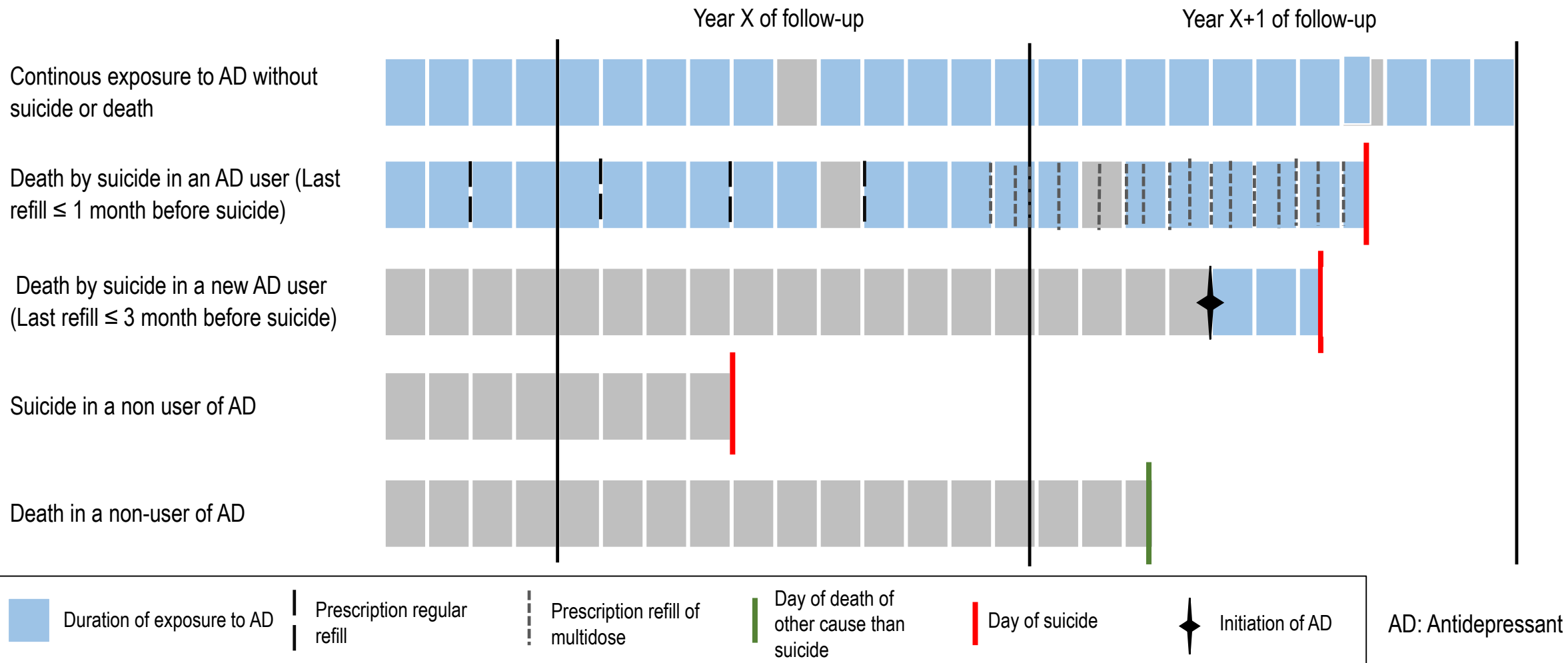

Supplement: Supplementary Material 1 — Figure visualizing antidepressant exposure patterns applied in a retrospective cohort study of adults aged 75 years and over. [file Data_Sheet_1.PDF]
