# Supplementary material for: Antidepressant Use and Suicide Rates in Adults Aged 75 and Above: A Swedish Nationwide Cohort Study
Source: Front Public Health. 2021 Feb 19;9:611559. doi: 10.3389/fpubh.2021.611559 (PMC7933212; doi:10.3389/fpubh.2021.611559)
Supplement: Supplementary Material 2 — Calculations of the decomposition method. [file Table_1.DOCX]

**Supplementary Material 2. Calculations of the decomposition method**

The overall suicide rate is equal to the summing of each treatment group’s suicide rate multiplied by the proportion of person-years spent in that group at time t.

If we denote the population composition for each treatment group as w*_i_*(*t*)= PY*_i_*(*t*)/PY(*t*), this gives the suicide rate in year t as

$r\left( t \right)= \sum_{i} r_{i}(t)w_{i}(t)$ (a)

Where the suicide rate r_i_ for each user-group *i*, is denoted by the number of suicides n_i_ divided with then number of patient years PYi(t) in time period t: r_i_(t) = n_i_(t)/PY_i_(t).

The change over time in suicide rates is derived by derivation of equation a with respect to time

$dr(t)/dt =\sum_{i} {[dr}_{i}(t)/dt w_{i}(t)+r_{i}\left( t \right) dw_{i}(t)/dt]$ (b)

Equation b indicates that the contribution by each user-group *i* to the overall change consists of two components. The first component is the change in the suicide rate caused by changes in the treatment-specific suicide rate. The second component is the changes in the population composition.

Each user-groups total contribution to the change in total overall suicide rates is the sum of the above two components.

The application of the decomposition technique in men is illustrated above.

| **Characteristics** | **Formula** | **Men  No antidepressant** | **Men AD user** |
| --- | --- | --- | --- |
| **2007-2008** | | | |
| **Total number of person years 2007/2008** | PY(07/08) | 1 083 153 | |
| **Person-years [%]** | PY*_i_*(07/08) [w*_i_*(07/08)] | 1 025 564 [**94.7**] | 57 589 [**5.3**] |
| **Number of suicides** | n*_i_*(07/08) | 183 | 35 |
| **Suicide rate per 100 000** | r*_i_*(07/08) | **17.8** | **60.8** |
| **2013-2014** | | | |
| **Total number of person years (%)** | PY(13/14) | 787 564 | |
| **Person-years [%]** | PY*_i_*(13/14) [w*_i_*(13/14)] | 723 378 [**91.9**] | 64 185 [**8.1**] |
| **Number of suicides** | n*_i_*(13/14) | 198 | 47 |
| **Suicide rate per 100 000** | r*_i_*(13/14) | **27.4** | **73.2** |
| **Derivation of decomposition components** | | | |
| **Mean of proportions** | A: (Ci(07/08)+Ci(13/14) /2) | (94.7/100+91.9/100) /2 = 0.933 | (5.3/100+8.1/100) /2= 0.067 |
| **Difference in suicide rates** | B: Si(2013/2014 – 2007/2008) | 27.4 – 17.8 = 9.6 | 73.2 – 60.8 = 12.4 |
| **Difference in proportions** | C: Ci(13/14) - Ci(07/08)) | 91.9/100 - 94.7/100 = -0.028 | 8.1/100 - 5.3/100 = 0.028 |
| **Mean of suicide rates** | D: (Si(2013/2014) + Si (2007/2008) /2) | (17.8 + 27.4) /2 = 22.6 | (60.8 + 73.2) /2 = 67.0 |
| **Change in suicide rate caused by changes in treatment-specific suicide rate** | A*B | 8.95 | 0.83 |
| **Change in suicide rate caused by changes in population composition** | C*D | -0.64 | 1.9 |
| **Total contribution to change in suicide rate** | A*B+C*D | 8.31 | 2.73 |
