# Supplementary material for: Antidepressant Use and Suicide Rates in Adults Aged 75 and Above: A Swedish Nationwide Cohort Study
Source: Front Public Health. 2021 Feb 19;9:611559. doi: 10.3389/fpubh.2021.611559 (PMC7933212; doi:10.3389/fpubh.2021.611559)
Supplement: Supplementary Material 3 — Biannual suicide rates per 100,000 (95% CI) by antidepressant treatment group for individuals aged 75 years and over in Sweden from 2007 and 2014. [file Table_2.DOCX]

**Supplementary material 3**

**Table 1. Bi-annual suicide rates per 100 000 (95% CI) by antidepressant treatment group for individuals aged 75 years and over in Sweden from 2007 and 2014^a^**

| **Period** | **2007-2008** | **2009-2010** | **2011-2012** | **2013-2014** | **Average bi-annual suicide rate** |
| --- | --- | --- | --- | --- | --- |
| **Total** | | | | | |
| **No antidepressant use** | 10.5 (9.3-11.7) | 11.4 (10.2-12.6) | 12 (10.7-13.4) | 16.8 (15.1-18.6) | 12.7 (8.2-17.2) |
| **Antidepressant use** | 28.7 (25.6-31.9) | 33 (29.3-36.7) | 36.7 (32.6-40.8) | 35.5 (31.8-39.2) | 34.1 (27.8-39.1) |
| Single use of SSRI | 20.8 (18.4-23.1) | 24.4 (21.7-27.1) | 24.7 (22-27.5) | 29.3 (26.2-32.5) | 24.7 (19.3-30.3) |
| Single use of mirtazapine | 36.9 (32.8-41) | 40.7 (36.2-45.2) | 44.4 (39.5-49.3) | 44.3 (39.8-48.8) | 41.6 (35.9-47.2) |
| Single use of other antidepressant | 58.7 (52-65.4) | 59.2 (52.5-65.8) | 70.6 (62.8-78.4) | 39.3 (35.2-43.4) | 57.0 (36.3-77.6) |
| …Use of ≥ 2 antidepressants | 76 (67.4-84.6) | 54.6 (48.6-60.7) | 94.1 (83.6-104.7) | 35.8 (32-39.5) | 65.1 (24.8-105.5) |
| **Men** | | | | | |
| **No antidepressant use** | 17.8 (15.5-20.2) | 18.2 (15.9-20.6) | 20.4 (17.7-23.1) | 27.4 (23.8-30.9) | 20.9 (13.9-28.0) |
| **Antidepressant use** | 60.8 (52.7-68.8) | 84 (72.8-95.1) | 65.9 (56.9-74.9) | 73.2 (64-82.4) | 71.0 (55-87.0) |
| Single use of SSRI | 43 (37.3-48.6) | 57 (49.4-64.7) | 37.6 (32.5-42.7) | 53.6 (46.9-60.3) | 47.8 (33.4-62.2) |
| Single use of mirtazapine | 85.7 (74.3-97.1) | 110.5 (95.8-125.2) | 85.4 (73.8-96.9) | 93.8 (82.1-105.6) | 93.8 (75.1-112.6) |
| Single use of other antidepressant | 84.3 (73.1-95.4) | 22.1 (19.2-25) | 146.7 (126.9-166.5) | 110.5 (96.6-124.4) | 90.9 (7.3-174.5) |
| …Use of ≥ 2 antidepressants | 178.3 (154.5-202) | 166.4 (144.2-188.5) | 149.7 (129.3-170.1) | 78.1 (68.3-87.9) | 143.1 (71.7-214.6) |
| **Women** | | | | | |
| **No antidepressant use** | 4.7 (3.7-5.7) | 6 (4.8-7.2) | 5.5 (4.3-6.7) | 8.6 (7-10.2) | 6.2 (3-5-8.9) |
| **Antidepressant use** | 16.2 (12.7-19.8) | 12.9 (10.1-15.6) | 25 (20-30.1) | 19.9 (16.2-23.7) | 18.5 (10.2-26.8) |
| Single use of SSRI | 12.1 (9.6-14.7) | 11.6 (9.3-14) | 19.7 (15.8-23.6) | 19.6 (15.8-23.3) | 15.8 (8.6-22.9) |
| Single use of mirtazapine | 17.2 (13.5-21) | 11.5 (9.2-13.9) | 26.7 (21.4-32) | 22.3 (18.2-26.4) | 19.4 (9.0-29.9) |
| Single use of other antidepressant | 49.7 (39.1-60.2) | 20.1 (16-24.3) | 39.7 (31.7-47.7) | 9.3 (7.6-11.1) | 29.7 (1.0-58.9) |
| …Use of ≥ 2 antidepressants | 37.4 (29.3-45.4) | 9.6 (7.6-11.5) | 73.1 (58.4-87.8) | 19.7 (16-23.5) | 34.9 (1.0-79.4) |

^a^Excluding persons using tricyclic antidepressants and no other AD (N=46 244)
